# Gut microbiota and metabolites: emerging prospects in the treatment of non-small cell lung cancer

**DOI:** 10.3389/fimmu.2025.1638942

**Published:** 2025-11-05

**Authors:** Jing-Mian Jiao, Chen-Guang Liu, Dan Zang, Jun Chen

**Affiliations:** Department of Oncology, The Second Hospital of Dalian Medical University, Dalian, China

**Keywords:** non-small cell lung cancer (NSCLC), gut microbiota, metabolites, treatment, prognosis

## Abstract

Non-small cell lung cancer (NSCLC) is the most prevalent form of lung cancer, accounting for approximately 85% of all cases, and is associated with a poor prognosis. Despite significant advancements in treatment modalities, therapeutic efficacy remains suboptimal, underscoring the urgent need for novel strategies. In recent years, increasing attention has been directed toward the pivotal role of gut microbiota-host interactions in the treatment of NSCLC. This review systematically examines the influence of current NSCLC therapies on gut microbiota and metabolism, explores the relationship between the microbiome and therapeutic response, and highlights the critical functions of probiotics, microbial metabolites, fecal microbiota transplantation (FMT), and dietary interventions in NSCLC management. By elucidating the mechanisms through which gut microbiota and their metabolites modulate treatment efficacy, we investigate the potential of exogenous interventions targeting the gut ecosystem to enhance therapeutic outcomes and mitigate adverse effects. Modulating the intestinal microbiota represents a promising clinical avenue and offers a new frontier for the development of future NSCLC treatment strategies.

## 1 Introduction

The human microbiome comprises a diverse and dynamic community of microorganisms—including bacteria, fungi, viruses—their genetic material, and metabolic byproducts. The resident microbiota is an essential component of host health and homeostasis (1). Most microbiome research to date has focused on bacterial populations, which constitute a major proportion of these resident microbes (2). In the gut, Bacteroidetes, Firmicutes, Proteobacteria, and Actinobacteria dominate the bacterial composition (3–5). The gut microbiota plays a pivotal role in regulating host immunity and metabolism through the production of numerous metabolites that function as signaling molecules and metabolic substrates, linking dysbiosis with inflammation and tumorigenesis (6–8).

The cross-link between gut microbiota and lung cancer is a complex multifactorial relationship (5). Studies have shown that in patients with lung cancer, the abundance of Bacteroidetes, Fusobacteria, Cyanobacteria, and Spirochaetes increases in both pulmonary and intestinal microbiomes, while Firmicutes are significantly reduced (4, 9). Research on both gut and respiratory tract microbiota has revealed notable dysregulation in NSCLC, which is further associated with distant metastasis (DM) (10). The pathogenic contribution of the gut microbiome and its specific metabolites to NSCLC lies in their modulation of chronic inflammation and immune dysregulation (11). A study combining serum metabolomics and fecal microbiome profiling identified potential biomarkers in patients with early-stage NSCLC. The metabolomic analysis revealed elevated levels of sphingolipids (e.g. D-erythrosphingosine 1-phosphate, palmitoylsphingomyelin), fatty acyls (e.g., Avocadyne 1-acetate, 12(S)-HETE, 20-carboxyleukotriene B4, thromboxane B3, 6-keto-prostaglandin F1α, decanoic acid, tetracosanoic acid), and glycerophospholipids in these patients (12).

Substantial progress has been made in NSCLC treatment in recent years, particularly in early screening, minimally invasive procedures, radiotherapy, targeted therapies, and immunotherapy. These advances have significantly improved patient survival rates (13). However, several challenges persist, including the emergence of drug resistance, treatment-associated toxicity, high costs, underrepresentation of minority groups in clinical trials, and limited access to diagnostic and therapeutic resources. These issues highlight the urgent need for novel therapeutic strategies to expand treatment options (14). Manipulating the gut microbiota has emerged as a promising strategy to enhance NSCLC treatment efficacy. Microbiome modulation may augment immunotherapeutic responses, mitigate adverse treatment effects such as microbial dysbiosis, and serve as a predictive biomarker for personalized therapy and disease prevention (15).

This review provides a comprehensive overview of how various NSCLC treatment modalities influence the gut microbiota and its metabolic profile. It further emphasizes the mechanisms and potential of microbiota-targeted interventions in improving clinical outcomes. Understanding the intricate relationship between gut flora, its metabolites, and NSCLC treatment holds substantial theoretical and clinical relevance, offering new insights into disease pathogenesis and therapeutic innovation.

## 2 Alterations in gut microbiota and metabolism induced by standard NSCLC treatments

Intestinal microbiota not only directly participates in the regulation of host tumor immunity but also indirectly influences tumor progression and therapeutic outcomes through the production of metabolic products. Within the gut microbiome, metabolites such as short-chain fatty acids disrupt the intestinal barrier, thereby affecting both innate and adaptive immunity, which triggers and exacerbates systemic immune dysregulation. Chronic inflammation, immune imbalance, and the activation of cancer-associated signaling pathways by specific bacterial strains are considered key mechanisms underlying the ecological imbalance and the immunosuppressive microenvironment (16). In patients with non-small cell lung cancer (NSCLC), the evolution and composition of the gut microbiota are closely linked to the efficacy and adverse effects of treatments such as chemotherapy, radiotherapy, immunotherapy, and targeted therapy, with microbial changes playing a crucial role in predicting both therapeutic outcomes and treatment-related side effects (17, 18).

### 2.1 Chemotherapy

**Figure 1:**
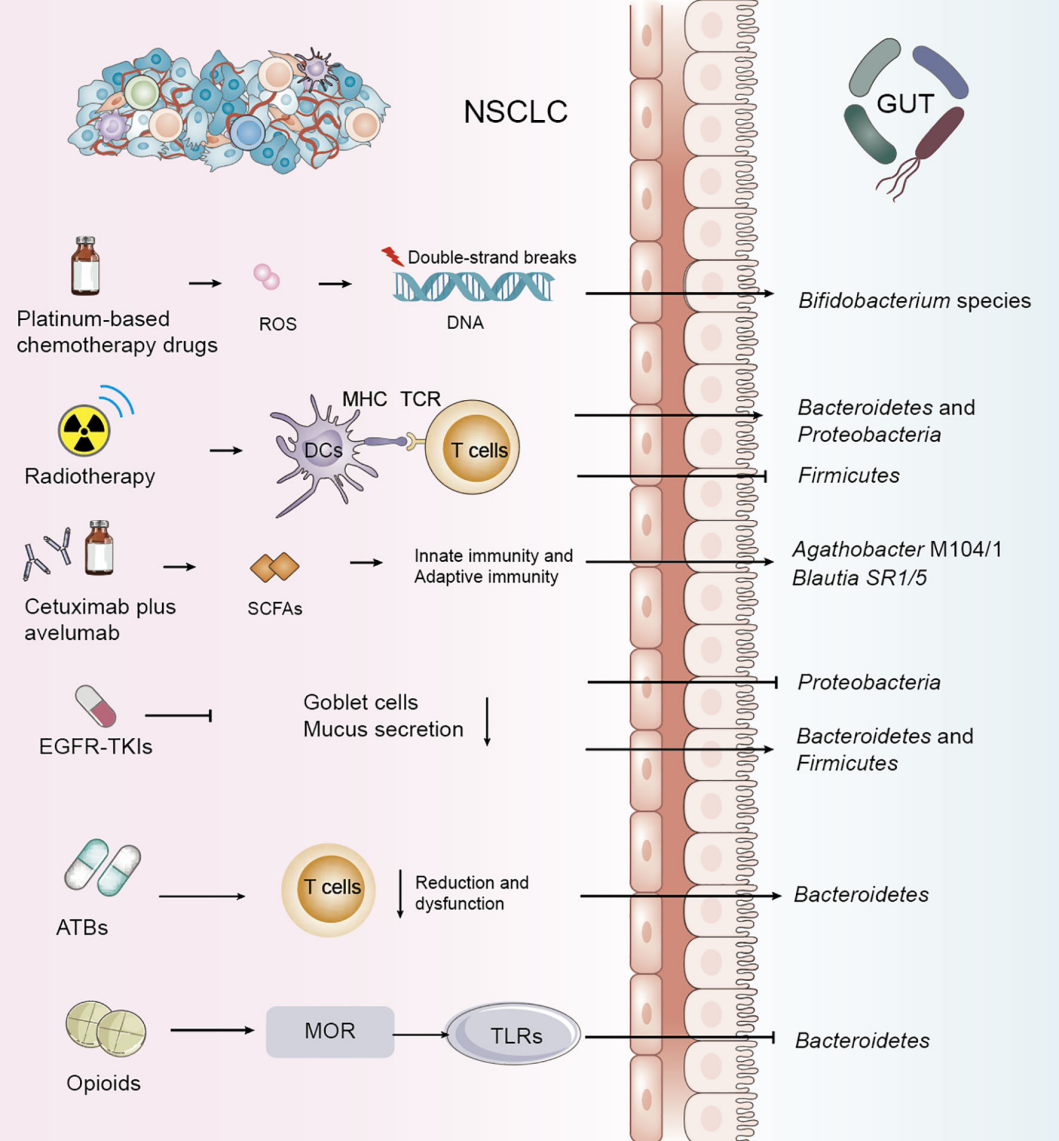
Impact of NSCLC treatments and adjunctive therapies on gut microbiota and metabolism. Common treatment modalities for non-small cell lung cancer (NSCLC), exert direct or indirect effects on the gut microbiota. These alterations highlight the dynamic and reciprocal relationship between NSCLC therapies and gut microbiota composition.

Chemotherapeutic agents are known to alter the composition of the gut microbiota, and several studies have confirmed significant shifts in specific microbial populations during NSCLC chemotherapy (19–23). Platinum-based drugs exert antitumor effects by inhibiting DNA replication and targeting cellular membranes and mitochondria, forming intra-strand platinum-DNA adducts that lead to double-strand breaks (DSBs) (24). These agents may exert their tumor-suppressive effects through microbiota-dependent pathways, with their efficacy partly relying on the intra-tumoral generation of reactive oxygen species (ROS), which decreases in response to reduced DNA damage. Particularly, an increase in *Bifidobacterium* species has been observed during platinum-based treatment of NSCLC (**Table 1**, **Figure 1**) (25). Pemetrexed, a multitargeted antifolate, inhibits several folate pathway enzymes—thymidylate synthase, dihydrofolate reductase, and glycinamide ribonucleotide formyltransferase—which are involved in purine and pyrimidine nucleotide synthesis for DNA and RNA production (26–28). In mouse models, pemetrexed treatment altered the gut microbiota, significantly increasing the relative abundance of *Enterococcaceae, Lactobacillaceae*, and *Streptococcaceae* (19). Paclitaxel was found to decrease the overall abundance of gut microbiota in lung cancer-bearing mice, with a significant disruption in the *Bacteroidetes/Firmicutes* ratio (p < 0.01) (29).

**Table 1:** Alterations in gut microbiota and metabolism induced by NSCLC treatments.

Treatments	Interventions	Mechanism	Gut bacteria/metabolites	Outcome	References
Chemotherapy	Platinum-based drugs	relying on the intra-tumoral generation of reactive oxygen species(ROS)decreases in response to reduced DNA damage	Bifidobacterium species	increasing	(22)
Radiotherapy	CCRT	Regulation of dendritic cell antigen presentation and radiation-induced anti-tumor immune response	Bacteroidetes and Proteobacteria Firmicutes	increasing decreasing	(30)
Immunotherapy	cetuximab plus avelumab	Butyrate, through the activation of innate and adaptive immunity, is a key modulator of host immune reactivity under pathological conditions	Agathobacter M104/1 and Blautia SR1/5	increasing	(34)
Targeted Therapy	EGFR-TKIs	Reduction in the number of goblet cells leads to impaired mucus secretion and disruption of the mucosal barrier	Proteobacteria Firmicutes and Bacteroidetes	decreasing increasing	(46)
Adjunctive Treatments	Antibiotics	impaired T-cell immunity	Bacteroidetes	decreasing	(56)
	Analgesic Drugs	activation of the mu-opioid receptor (MOR) by opioid ligands has been found to disrupt tight junction protein (ZO-1) coordination through activation of toll-like receptors (TLR) 2 and 4, thereby affecting intestinal epithelial integrity	Bacteroidetes	decreasing	(63)

### 2.2 Radiotherapy

For patients with locally advanced NSCLC, concurrent chemoradiotherapy (CCRT) is a standard treatment. Recent studies have shown that alpha diversity of gut microbiota is significantly associated with therapeutic response, indicating that dysbiosis is a key environmental factor affecting prognosis. One study explored dynamic changes in gut microbiota and their predictive value for progression-free survival (PFS) following CCRT in NSCLC patients. It was observed that the abundance of *Bacteroidetes* and *Proteobacteria* increased, while *Firmicutes* decreased after treatment. Patients with longer PFS demonstrated significantly greater diversity in fungi, archaea, and viruses compared to those with shorter PFS. Key metabolic pathways affected included fatty acid metabolism, arginine biosynthesis, lipopolysaccharide biosynthesis, and ascorbate and aldarate metabolism (**Table 1**, **Figure 1**) (30, 31).

### 2.3 Immunotherapy with immune checkpoint inhibitors

#### 2.3.1 Immunotherapy

Programmed death ligand 1 (PD-L1) signaling plays a role in maintaining gut mucosal tolerance. However, the direct link between host microbiota and tumor PD-L1 expression remains unclear (32). Treatment of NSCLC with immune checkpoint inhibitors (ICIs), including anti–PD-1/PD-L1 and anti–CTLA-4 agents, induces significant shifts in gut microbiota and metabolic profiles. Metagenomic sequencing has revealed correlations between microbial characteristics and clinical features such as PFS and PD-L1 expression levels (33). One study characterized the microbiome from bronchoscopic tumor biopsies of NSCLC patients undergoing ICIs therapy using 16S rRNA sequencing, revealing high levels of *Firmicutes*, *Bacteroidetes*, and *Proteobacteria* (29) The phase II CAVE-LUNG clinical trial examined the effects of cetuximab plus avelumab in chemotherapy-refractory NSCLC patients and identified increased expression of *Agathobacter* M104/1 and *Blautia* SR1/5 after treatment (P=0.016 and P=0.0008, respectively) (**Table 1**, **Figure 1**) (34). One study analyzing gut microbiota in NSCLC patients receiving atezolizumab-based immunotherapy found that microbial composition and diversity changed over the course of treatment, aiding the development of predictive biomarkers and microbiota-based biotherapeutics. Genera such as *Clostridium, Lachnospiraceae*, and *Ruminococcaceae* have been identified as potential biomarkers of therapeutic response (35). The therapeutic efficacy of ICIs is markedly enhanced in patients demonstrating durable clinical benefit as well as in those with tumors expressing PD-L1, with numerous studies underscoring this correlation (36).

#### 2.3.2 irAEs

The complex interplay among the tumor microenvironment, gut microbiota, host factors, and responses to immune checkpoint inhibitors (ICIs), as well as the development of ICI-related immune-related adverse events (irAEs), remains largely unknown. Emerging evidence suggests that the gut microbiota may play a critical role in modulating tumor responses to ICIs (37). ICIs exert their therapeutic effect by activating T cells, a process frequently accompanied by autoimmune phenomena collectively termed “immune-related adverse events (irAEs).” These irAEs exhibit pleiotropic manifestations that can affect virtually any organ system, including the skin, colon, endocrine glands, joints, heart, and lungs. Importantly, the gut microbiota has been proposed as a potential biomarker for predicting irAEs. A recent study integrating gut microbiota metabolites, molecular modeling, and species-level variation identified signatures that shaped long-term therapeutic efficacy and adverse outcomes in lung cancer survivors. Patients who developed irAEs exhibited reduced abundance of *Roseburia faecis*, *Roseburia intestinalis*, *Bacteroides stercoris*, *Lactobacillus mucosae*, and *Akkermansia muciniphila* (38). These findings suggest that the gut microbiome may serve both as a risk factor and a protective factor for irAEs. Notably, checkpoint inhibitor-induced colitis remains the most frequently reported irAE (39).

The gut microbiota has also been demonstrated to play a pivotal role in shaping the tumor immune microenvironment, thereby influencing the efficacy of ICIs (40). Alterations in gut microbial composition and function are associated with an increased risk of irAEs, and predictive models of such risks have been developed. For example, advanced machine learning approaches have been employed to identify gut microbial signatures capable of predicting irAE occurrence (39). A random forest classifier constructed from 14 microbial features exhibited strong discriminatory power between irAE and non-irAE groups. Functional analyses revealed that the gut microbiota of non-irAE patients was characterized by increased menaquinone biosynthesis, accompanied by upregulation of the rate-limiting enzymes *menH* and *menC*. Targeted metabolomic profiling further confirmed significantly higher serum menaquinone levels in non-irAE patients compared to those who developed irAEs (41). Collectively, these findings highlight the dual role of gut microbiota in shaping both therapeutic efficacy and toxicity of ICIs. Future research integrating metagenomic, metabolomic, and functional analyses will be critical to unravel the mechanistic underpinnings of these associations and to enable the development of microbiome-based biomarkers and therapeutic strategies for optimizing NSCLC treatment outcomes.

### 2.4 Targeted therapy

Targeted therapy using tyrosine kinase inhibitors (TKIs) has become a cornerstone in NSCLC treatment alongside chemo- and radiotherapy (42). Studies examining the gastrointestinal microbiome in patients with Epidermal Growth Factor Receptor (EGFR)-wild-type versus EGFR-mutant NSCLC revealed a predominance of *Proteobacteria*, implicating its role in disease mechanisms (43). In EGFR-mutant patients treated with EGFR-TKIs, lower levels of *Proteobacteria* and higher levels of *Bacteroidetes* and *Firmicutes* were observed (44). Despite the efficacy of second-generation TKIs such as afatinib, clinical trials have reported grade ≥3 diarrhea in over 25% of patients, with about 15% discontinuing treatment due to severe diarrhea, compromising therapeutic outcomes (45). Mouse models have shown significant increases in *Peptostreptococcus, Staphylococcus, Escherichia-Shigella*, and *Akkermansia* following afatinib treatment (**Table 1**, **Figure 1**) (46). A study investigating the gut microbiota profile in fecal samples from a lung-specific conditional EGFR mutant transgenic mouse model of lung tumorigenesis demonstrated that *Lactobacillus*, a genus of bacteria known for producing short-chain fatty acids (SCFAs), may serve as a predictive factor for tumor initiation and progression in EGFR mutation-induced lung adenocarcinoma models (47).

## 3 Effects of adjunctive treatments on gut microbiota and metabolic alterations

### 3.1 Antibiotics

Antibiotics have a significant impact on gut microbiome health, often leading to dysbiosis or the proliferation of harmful flora that can undermine the effectiveness of therapies such as immunotherapy for NSCLC. Antibiotic use has been associated with poor responses to combination therapies, including immunotherapy (48–54). Analysis of bacterial phyla has shown that antibiotic treatment for up to 4 weeks prior to immunotherapy increases the abundance of *Bacteroidetes*. Systemic use of antibiotics has been linked to an increased *Bacteroidetes/Firmicutes* ratio, correlating with poorer immunotherapy outcomes (55). Broad-spectrum antibiotic-associated gut microbiome dysbiosis, occurring in patients treated with long-term antibiotics, leads to impaired T-cell immunity (**Table 1**, **Figure 1**) (56). A study sequencing 16S ribosomal DNA (rDNA) from 69 fecal samples of advanced NSCLC patients prior to immune checkpoint blockade (ICB) therapy revealed that antibiotic use was significantly associated with a decrease in gut microbiota diversity (57). An analysis of bacterial diversity and differential abundance of fecal samples from NSCLC patients treated with anti-PD-1/PD-L1 antibodies showed that feces from patients not treated with antibiotics were enriched in Clostridium perfringens, especially within the *Rumatococcaceae*, *UCG13*, *Clostridium* spp., and *Agathrobacterium* spp. families. In contrast, feces from patients who received antibiotics were enriched in *Hungatella* (48). Plasma citrulline, a marker of intestinal barrier function, decreases early in NSCLC patients treated with nab-paclitaxel following antibiotic use, affecting citrulline metabolism and, consequently, intestinal microbiome metabolism (58). Vancomycin preferentially targets Gram-positive bacteria, including butyrate-producing species, reducing short-chain fatty acid (SCFA) concentrations in fecal and tissue samples. Vancomycin also eliminates two major families of SCFA-producing *Clostridia*: *Ruminococcaceae* and *Eubacteriaceae* (59). However, further studies are required to determine whether antibiotic use directly alters intestinal flora and metabolism, and to explore the mechanisms by which antibiotic use may reduce the efficacy of NSCLC treatments by modulating intestinal flora and metabolism.

### 3.2 Analgesic drugs

The gut microbiome plays an important role in modulating visceral pain, and recent evidence suggests that it may also be involved in various types of chronic pain, such as inflammatory pain, headaches, neuropathic pain, and opioid tolerance (60). Morphine and other opioids disrupt the gut barrier, alter gut flora and metabolism, and impair function by inhibiting mucus and bicarbonate secretion, disrupting muscle coordination, and increasing the risk of bacterial translocation (61). Long-term morphine use has been shown to significantly alter the gut microbiome, promoting the growth of Gram-positive pathogens while reducing biliary isolates (62). Additionally, activation of the mu-opioid receptor (MOR) by opioid ligands has been found to disrupt tight junction protein (ZO-1) coordination through activation of toll-like receptors (TLR) 2 and 4, thereby affecting intestinal epithelial integrity (**Table 1**, **Figure 1**) (63). Therefore, the rational use of analgesic drugs is also critical in the treatment of NSCLC, and it has been shown that analgesic drugs alter the gut microbiota, which in turn is detrimental to the treatment of NSCLC.

### 3.3 Traditional Chinese medicine as adjunctive treatment

The fundamental theory of Traditional Chinese Medicine (TCM) for treating NSCLC focuses on restoring balance through the principles of ‘strengthening the body’ and ‘eliminating evils’ when the body’s immunity is weak, and the tumor’s growth is overly strong. “Strengthening the body” refers to enhancing the body’s anti-cancer immunity, while “eliminating evil” directly inhibits the growth, proliferation, invasion, and migration of tumor cells. The clinical manifestations of NSCLC are often characterized by lung qi and lung yin deficiency. TCM’s core therapeutic paradigm for lung cancer revolves around the concepts of **‘**lung qi deficiency’ and ‘qi-yin deficiency**’**, with an emphasis on “supporting the positive and curtailing the negative” (4, 64). It has been investigated that combination therapy with monoclonal antibodies remodeled the composition of the gut microbiota and increased the number of SCFAs-producing bacteria Muribaculum to sensitize the antitumor effects of anti-PD-1 therapy and restore the microbial composition of fecal samples from those who did not respond to anti-PD-1 therapy (65). Polysaccharides derived from Spirulina have been shown to increase the abundance of Lactobacillus, Allobaculum, Alloprevotella, and Olsenella, while reducing Bacteroides and Acinetobacter levels. These effects may be linked to the inhibition of lung cancer in mice (66). Fuzi-Li zhong pill (FLP) is a well-validated TCM formula that has long been used in China for gastrointestinal disease and adjunctive therapy for depression (67). Lateralis Radix Praeparata (Fuzi), a traditional Chinese herb, is known for its relatively low toxicity and has been found to improve gut dysbiosis in NSCLC, decreasing the abundance of Proteobacteria while increasing that of Firmicutes (68, 69). Si junzi Decoction (SJZD) is a traditional Chinese medicine formula widely used in the treatment of gastrointestinal disorders. Despite its proven effectiveness, the precise mechanisms by which SJZD operates remain incompletely understood (70). A study evaluating the efficacy of SJZD on quality of life, hematological parameters, and modulation of gut flora in post-surgical NSCLC patients demonstrated that SJZD had a favorable effect on increasing microbial abundance and diversity, promoting probiotic microorganisms, and modulating microbial functions (71).

## 4 Beneficial intervention strategies for improving NSCLC treatment outcomes

### 4.1 Probiotic supplementation

**Figure 2:**
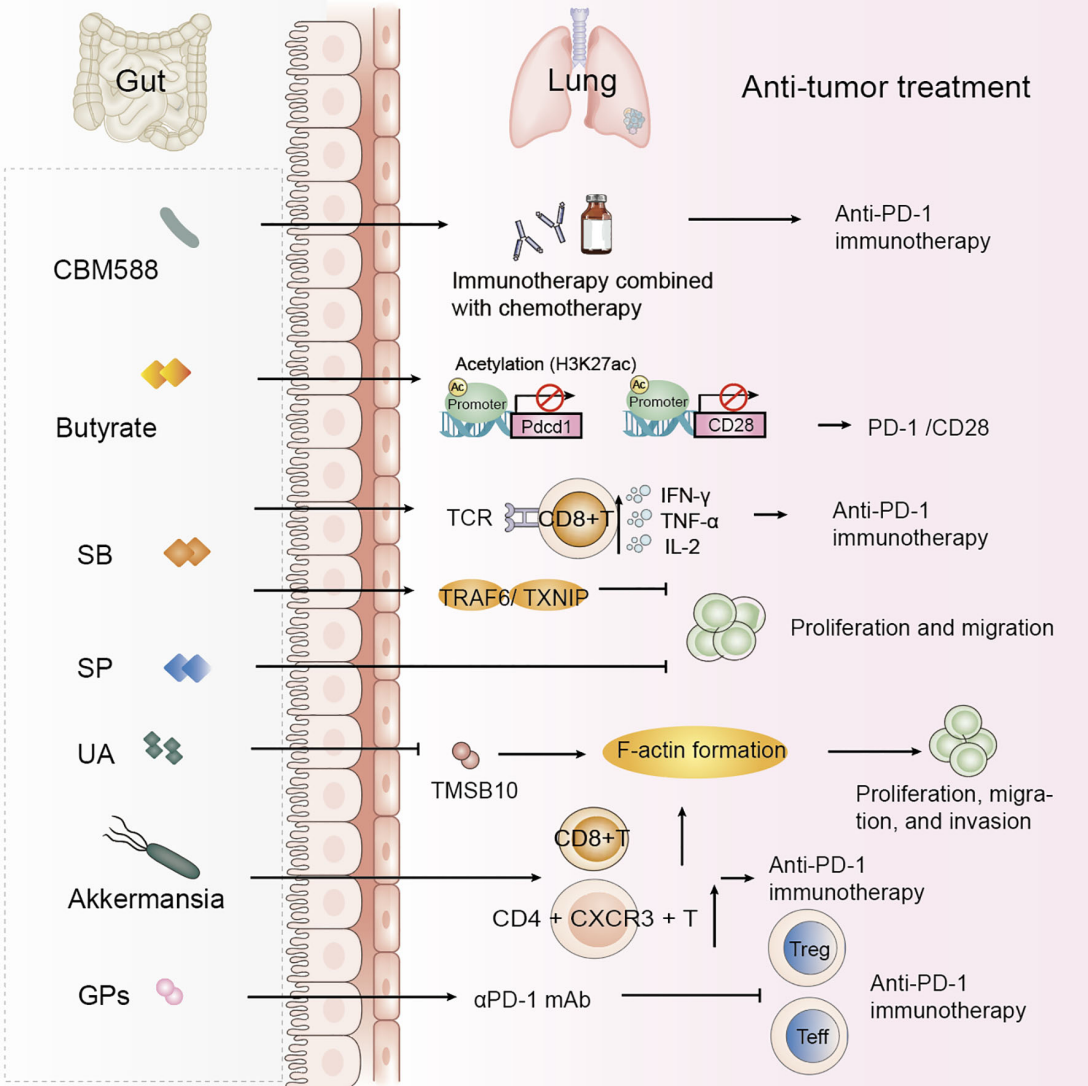
Gut microbiota-derived metabolites and probiotics improve NSCLC treatment efficacy. Specific gut microbiota and their metabolites modulate host immune responses and impact the efficacy of anti-tumor therapies in NSCLC. These findings highlight the therapeutic potential of microbiota-derived interventions in improving therapeutic outcomes in NSCLC. SB, Sodium butyrate; SP, Sodium propionate; UA, Urolithin A; GPs, Ginseng polysaccharides.

Probiotics have previously been shown to alter gut microbiota composition, thereby influencing cancer treatment outcomes. A study on the anticancer potential of probiotics suggests that gut probiotics exert tumor-suppressive effects through the gut-lung axis microecological regulation (72). Probiotics have been positively associated with overall survival (OS) and progression-free survival (PFS) in NSCLC patients treated with ICIs (73). Further research has shown that probiotics did not affect PFS but identified two dynamic types of gut flora during immunotherapy: one type exhibited the lowest relative abundance at the response time point, while the other showed the highest abundance at the response time point (74). Specific changes in intestinal flora, such as those induced by *Clostridium butyricum*, have been found to influence clinical outcomes in non-squamous NSCLC (NS-NSCLC) patients receiving bevacizumab combined with platinum chemotherapy, significantly reducing adverse events in patients (21). *Clostridium butyricum* MIYAIRI 588 (CBM588) has been shown to potentiate the efficacy of PD-1 blockade in NSCLC by modulating gut microbiota diversity and immune responses. CBM588 supplementation enhanced IL-10 secretion by lamina propria monocytes, improved intestinal homeostasis, and facilitated CD8^+^ T-cell activation, thereby helping to overcome resistance to immunotherapy (**Table 2**, **Figure 2**) (75, 76).

**Table 2:** Specific microbiota or metabolites exert positive effects on NSCLC treatment.

Gut bacteria/metabolites	Mechanism	Outcome message	References
CBM588	Enhancing the ability of CD8 T cells to secrete interferon gamma	Overcoming the resistance to PD-1 blockade	(75)
Butyrate	Increasing histone 3 lysine 27 acetylation (H3K27ac) in the promoter region of Pdcd1 and CD28 in human CD8 T cells, thereby promoting PD-1/CD28 expression	Enhancing the efficacy of anti-PD-1 therapy	(85)
SB	Modulating TCR signaling in cytotoxic CD8 T cells	Promoting the efficacy of anti-PD-1 immunotherapy	(86)
SB	activation of the TNF receptor-associated factor 6 (TRAF6)-thioredoxin-interacting protein (TXNIP) pathway	affecting the proliferation and migration of A549 cells	(88)
UA	inhibits F-actin formation by decreasing the protein level of TMSB10, thereby	inhibiting the proliferation, migration, and invasion of A549 cells.	(91)

Live biotherapeutic products (LBPs), as defined by the U.S. Food and Drug Administration (FDA), are biological agents intended for disease prevention, treatment, or cure (77). Recently, LBPs designed to modulate the gut microbiota, such as *Bifidobacterium lactis* and *Lactobacillus acidophilus*, have shown potential therapeutic benefits, including in colorectal cancer [NCT03072641]. Of particular relevance to NSCLC, *CJRB-101*—an LBP containing *Catenibacterium mitsuokai*—has shown antitumor activity in humanized NSCLC mouse models when combined with pembrolizumab. Mechanistically, *CJRB-101* reprograms M2 macrophages into M1 macrophages co-expressing CXCL9 and CXCL10, thereby enhancing CD8^+^ T-cell activation and augmenting antitumor immune responses (78).

### 4.2 Supplementation with metabolites

Characterizing the microbiota and metabolomic profiles of patients provides opportunities to target microbiota-derived metabolites that modulate the tumor microenvironment (TME) (79). These metabolites can influence NSCLC progression and shape the antitumor activity of host immune cells. For example, the gut microbiota generates short-chain fatty acids (SCFAs), which not only exert direct antitumor effects but also enhance immune system function (80).

#### 4.2.1 SCFAs

In immunotherapy for NSCLC patients, specific intestinal flora can enhance T-cell responses and activate anti-tumor immune mechanisms through metabolites to improve therapeutic efficacy (81, 82). Studies have demonstrated that the diversity of gut microbiota and short-chain fatty acids (SCFAs) is closely associated with the efficacy of immunotherapy (83). Metagenomic analyses reveal significant differences in metabolic pathways, with favorable responders exhibiting enhanced SCFA production. In murine models, fecal microbiota transplantation (FMT) and SCFA supplementation improved therapeutic outcomes by promoting effector T-cell activity within tumors (84).

Serum butyrate levels were positively correlated with the expression of programmed cell death-1 (PD-1) on circulating CD8 T cells and Vγ9 Vδ2 (Vδ2) T cells from NSCLC patients. Butyrate increased histone 3 lysine 27 acetylation (H3K27ac) in the promoter region of Pdcd1 and CD28 in human CD8 T cells, thereby promoting PD-1/CD28 expression and enhancing the efficacy of anti-PD-1 therapy (**Table 2**, **Figure 2**) (85). A study found a significant positive correlation between *Streptococcus* and CD8 T cell abundance, with the gut metabolite butyrate promoting the efficacy of anti-PD-1 immunotherapy by modulating TCR signaling in cytotoxic CD8 T cells (**Table 2**) (86). Butyrate supplementation also promotes the expression of anti-tumor cytokines in cytotoxic CD8 T cells through the T cell receptor (TCR) signaling pathway (**Figure 2**) (87). Another study highlighted the potential of sodium butyrate in inhibiting lung cancer cell growth, triggering apoptosis, inducing cell cycle arrest, and modulating the immune response through activation of peripheral blood CD4^+^ T cells while selectively inducing IFN-γ-R1 in peripheral blood NK cells and inhibiting CD8^+^ T cells and NK cells. Sodium butyrate’s mechanism of action in the tumor microenvironment and its effects on the immune system offer valuable insights into its potential as an adjuvant therapy for NSCLC (83). In mice, sodium butyrate was shown to affect the proliferation and migration of A549 cells through the activation of the TNF receptor-associated factor 6 (TRAF6)-thioredoxin-interacting protein (TXNIP) pathway, suggesting that sodium butyrate has an effective therapeutic effect on lung adenocarcinoma (**Table 2**) (88). Sodium propionate (SP) also inhibits the proliferation of lung cancer cells by inducing apoptosis and cell cycle arrest (11). Lower concentrations of circulating SCFAs in lung cancer patients may affect the host immune response (89). A study establishing associations between the gut microbiome and its metabolites, and SCFAs in NSCLC patients in early and brain metastatic stages suggests that specific forms of the gut microbiome and SCFAs may be of value in the treatment of NSCLC (90).

#### 4.2.2 Other metabolites

Urolithin A (UA), a natural compound produced by the gut microbiota through the metabolism of the polyphenols ellagitannin (ET) and ellagic acid (EA), has been found to inhibit epithelial-mesenchymal transition (EMT) in NSCLC cell lines. UA inhibits F-actin formation by decreasing the protein level of TMSB10, thereby inhibiting the proliferation, migration, and invasion of A549 cells. Contributing to the treatment of NSCLC (**Table 2**, **Figure 2**) (91). Baicalin is a metabolite that modulates the gut microbiota, exerting its effects through the regulation of short-chain fatty acids (SCFAs). Baicalin enhances the PD-1 (CD8^+^ T cell/Treg) balance and mitigates resistance to anti-PD-1 therapy (92).

The tryptophan–kynurenine (Trp–Kyn) metabolic axis, primarily regulated by indoleamine 2,3-dioxygenase 1 (IDO1) and tryptophan 2,3-dioxygenase (TDO), exerts a profound influence on dendritic cell antigen presentation and T-cell priming through tryptophan depletion and kynurenine accumulation. This metabolic reprogramming fosters an immunosuppressive tumor microenvironment that promotes immune tolerance and facilitates tumor immune evasion (93, 94). Among the major metabolic pathways implicated in NSCLC immunomodulation—including short-chain fatty acid (SCFA) and bile acid metabolism—the Trp–Kyn axis demonstrates the most direct mechanistic linkage to impaired antigen presentation and reduced T-cell activation. Moreover, tumor genotypes such as EGFR and KEAP1 mutations may further modulate IDO1/TDO activity, influencing immune resistance and responsiveness to immune checkpoint inhibitors (ICIs) (95). Consequently, targeting the Trp–Kyn pathway represents a promising strategy to enhance the efficacy of ICIs and overcome immune escape in NSCLC (96).

### 4.3 Fecal microbiota transplantation

To date, microbiome-metabolite (ME) research in oncology has primarily focused on the impact of gut microbiota composition on the efficacy of ICIs. Variations in the relative abundance of individual microbial strains and overall microbial diversity in patients undergoing ICI therapy appear to correlate with treatment outcomes (97). FMT has potential therapeutic benefits, particularly in converting non-responders to NSCLC treatment into responders. Fecal transplantation from anti-PD-1-responsive patients to non-responsive NSCLC patients increased treatment response rates without increasing toxicity (43, 57, 98). *Akkermansia muciniphila* (Akk) has been associated with the clinical benefits of ICIs in NSCLC patients, and the relative abundance of Akk may serve as a reliable biomarker for predicting good or poor prognosis in patients receiving PD-1 blockade immunotherapy, refining patient stratification in future studies (99). Akk enrichment modulation enhances immune responses through fecal microbiota transplantation in patients benefiting from immune checkpoint blockade (100). In animal experiments, the relative abundance of Akk predicted clinical response to PD-1 blockade in NSCLC patients. Mice receiving FMT, negative for *Fusobacterium* tachyzoites, exhibited tumor resistance to PD-1 blockade (15). RNA later preserved stool samples were collected from 65 pre-treatment (baseline) and post-treatment stage III/IV NSCLC patients treated with ICI and classified as responders or non-responders according to RECIST criteria. Mixed and individual responder and non-responder microbiota were transplanted into a gnotobiotic mouse model of lung cancer and treated with ICI, while patient fecal samples were subjected to 16S rDNA and RNA sequencing, which demonstrated that responding patients had a different microbial community structure (P=0.004) and a different bacterial transcriptome (PC2=0.03) at baseline. Taxa significantly enriched in responders included amplicon sequence variants (ASVs) belonging to the genera *Rumococcus, Akkermansia muciniphila*, and *E. faecalis.* Transplantation of mixed and individual responding microbiota into gnotobiotic mice reduced tumor growth compared to non-responding colonized mice after ICI use (P=0.023, P=0.019, P=0.008, respectively), showing an increased tumor CD8 ^+^ IFN γ ^+^ T-cells and CD4^+^ CXCR3^+^T-cells phenotype after ICI treatment. Responding mice were enriched with ASV belonging to the genera *Mycobacterium, Blautia, Akkermannia* and *E. faecalis* (101). However, many FMT studies have reported only limited methodological descriptions, details of mouse cohorts, and statistical methods. One study performed human-to-germ-free mouse FMT using fecal samples from NSCLC patients with pathological response or no response after neoadjuvant ICI treatment, which produced greater anti-tumor responses in R-FMT mice in combination with anti-PD-L1 therapy compared to NR-FMT, detailed study of the mouse microbiota after FMT using 16S rRNA amplicon sequencing, and use of models for the biological variables were classified and corrected, revealing that the most abundant taxa were shared between human inoculum and mice, although low abundance human taxa were more variable in post-FMT colonized mice. Multiple *Clostridium* spp. were also associated with tumor outcome in individual anti-PD-L1-treated R-FMT mice (102).

Research on Fecal Microbiota Transplantation (FMT) would benefit from well-designed large-scale studies that incorporate extensive metadata and standardized bio-sample collection. Such efforts are crucial to minimize noise in downstream multi-omics analyses and to ensure robust long-term follow-up to address potential safety concerns. The results of these studies could guide targeted experimental designs to explore the underlying mechanisms of FMT clinical outcomes and may ultimately lead to the personalized matching of donor and recipient characteristics to achieve optimal therapeutic success (103). Although FMT may be a promising therapeutic option, the risk of bacterial translocation (including antibiotic-resistant bacteria) and sepsis in patients remains a significant safety concern, and studies have been conducted that discuss sepsis due to FMT (104–106). The most feasible translational approach from whole-stool FMT toward safer and standardized microbiome-based therapies involves progressive refinement from complex donor-derived consortia to defined microbial communities and, ultimately, purified microbial metabolites with validated bioactivity (107, 108). Microbial ecosystem therapy (MET), comprising selected, well-characterized commensal strains, represents a key intermediate strategy that maintains ecological functionality while improving safety and reproducibility (109, 110). Preserving therapeutic efficacy along this continuum requires retention of crucial host–microbe interactions that regulate immune homeostasis (111). Therefore, future microbiota-targeted interventions should emphasize context-dependent functionality to achieve both safety and sustained immunotherapeutic benefit in NSCLC treatment.

### 4.4 Dietary interventions

Castalagin is a polyphenol that enhances resistance to PD-1. In their study, Messaoudene et al. reported that oral supplementation with polyphenol-rich berry camu-camu (CC; *Myrciaria dubia*) altered the gut microbial composition, leading to antitumor activity and a stronger anti-PD-1 response. Castalagin improved the CD8+/FOXP3+CD4+ ratio in the tumor microenvironment. Moreover, castalagin induced metabolic changes, resulting in an increase level of taurine-conjugated bile acids. *Ruminococcus*-rich NSCLC responders were found to be able to metabolize castalagin (112, 113). Patients should be advised to minimize animal meat intake and increase plant intake where possible, aiming for 30 plants per week. High fiber intake (>30 g/day) is thought to increase the chances of response to immunotherapy in NSCLC (114). Methionine regulates tumor immunity by modulating the activity of cyclic GMP-AMP synthase (cGAS), so the tumor immune response can be improved by controlling dietary methionine intake (115). Ginseng polysaccharides (GPs) are one of the most abundant constituents of ginseng, and GPs increase the antitumor response to αPD-1 mAb by inhibiting the metabolite *kynurenine/tryptophan* ratio, which contributes to the suppression of regulatory T-cells and the induction of Teff cells following combination therapy, enhancing the antitumor effects of immunotherapy (116). Specific diets directly or indirectly alter the intestinal flora and metabolism of NSCLC patients and increase the clinical efficacy of NSCLC.

In recent years, relevant clinical trials have been conducted around simulated fasting diets, high-fiber diets, nutritional supplements and other related trials, which have either maximized the therapeutic efficacy of NSCLC treatments or maximized the benefits for patients (117).

## 5 Role of gut microbiota and metabolites in NSCLC treatment and prognosis

### 5.1 Baseline microbiome as a predictive biomarker

As previously discussed, immunotherapy plays a crucial role in the treatment of NSCLC. Numerous studies have collected plasma and stool samples from patient cohorts prior to initiating immunotherapy, performing metabolomic and microbiome analyses (118, 119). The results obtained after enrichment are referred to as baseline microbiome characteristics. From a metagenomic perspective, enrichment of *Akkermansia* may be indicative of favorable prognosis in patients undergoing PD-1 blockade immunotherapy, offering potential for improved patient stratification in future studies (99). *Akkermansia muciniphila*, a mucin-degrading commensal bacterium, exemplifies the context-dependent functionality of microbiota-based interventions. Under conditions of adequate mucin renewal and minimal antibiotic disturbance, it reinforces gut barrier integrity and immune regulation (120, 121). However, in states of mucin depletion, chronic inflammation, or dysbiosis, its activity may shift from protective to deleterious, aggravating intestinal damage and immune dysregulation (122). These findings underscore the necessity of designing microbial consortia and derived metabolites that maintain host–microbe symbiosis and ensure both safety and therapeutic efficacy in diseases such as NSCLC (120, 122).

Additionally, metabolomic analyses suggest that patients enriched with baseline short-chain fatty acids (SCFAs) may derive long-term benefits from immunotherapy (90). The close link between microbiota and metabolites at baseline not only provides novel insights for clinical stratification but also opens new avenues for clinical translation (86). For instance, some studies have demonstrated SCFA enrichment via metabolomic profiling and verified the presence of microbiota producing SCFAs as a baseline marker for therapeutic efficacy. In parallel, other studies have enriched baseline microbiota and subsequently identified corresponding metabolites, confirming the therapeutic benefits of these metabolites at baseline enrichment (92).

In summary, baseline microbiome and metabolite characteristics have been explored in existing research, and many clinical trials have since emerged, contributing to this growing field.

### 5.2 Gut microbiota and metabolites in NSCLC clinical trials

Numerous clinical trials have focused on the role of gut microbiota and metabolites in various treatment modalities for NSCLC, examining their impact on treatment efficacy and prognosis. These studies underscore the importance of analyzing dynamic changes in the gut microbiota throughout the course of treatment, with specific microbial and metabolic alterations observed after a certain duration of therapy. By exploring the modulation of specific microbiota, these studies suggest that manipulating the microbiome could enhance NSCLC treatment outcomes. Such findings are poised to drive major breakthroughs in future NSCLC therapies.

**Figure 3:**
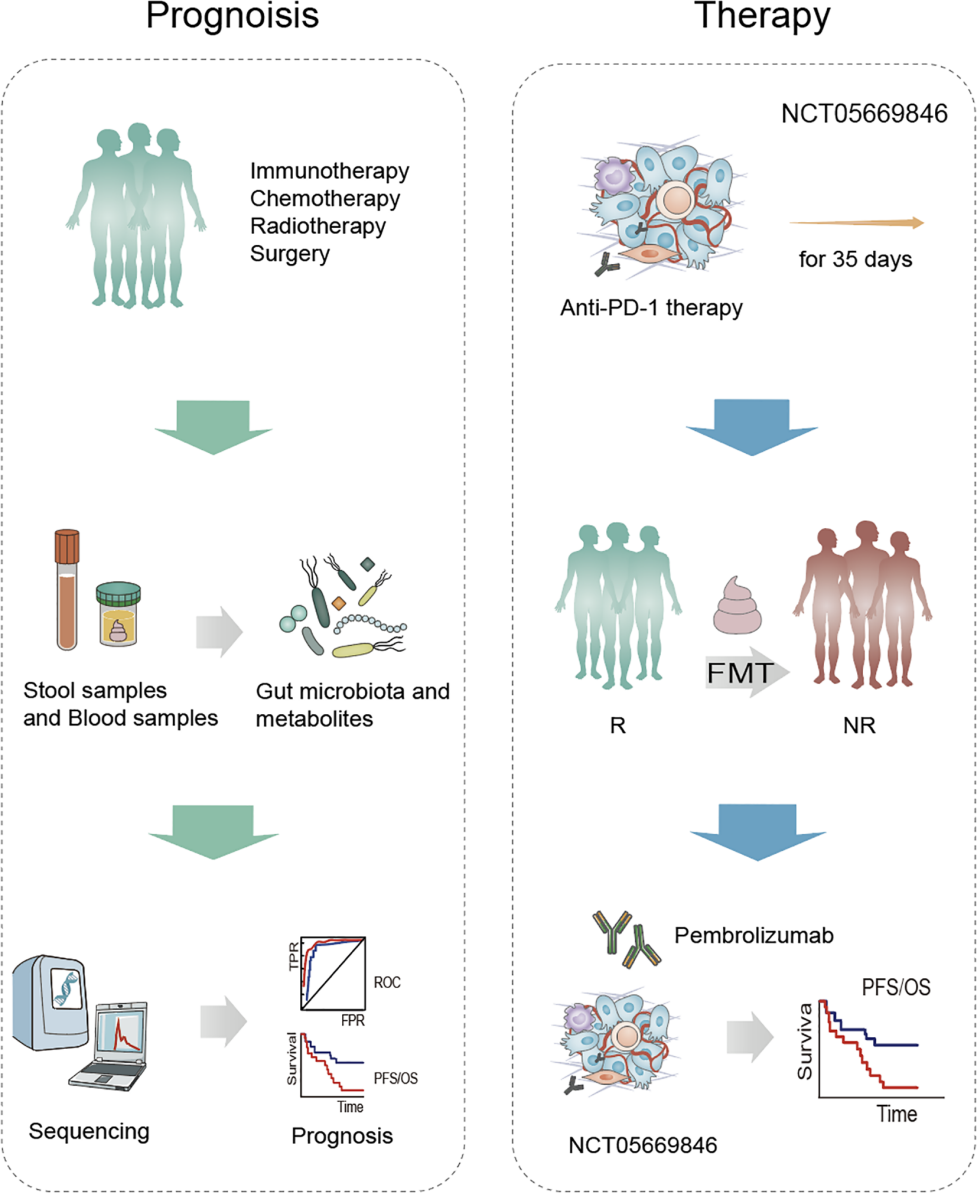
Microbiota-based strategies in the prognosis and treatment of NSCLC. Patients undergoing standard treatment modalities—such as immunotherapy, chemotherapy, radiotherapy, and surgery—provide stool and blood samples, from which gut microbiota and metabolites are analyzed. Subsequent sequencing and computational analysis identify microbial and metabolic biomarkers associated with treatment response and survival outcomes. As part of the clinical trial NCT05669846, patients receive anti-PD-1 therapy for 35 days. Fecal microbiota transplantation (FMT) is used to transfer gut microbes from responders (R) to non-responders (NR), followed by pembrolizumab treatment. This strategy aims to improve immune checkpoint inhibitor (ICI) efficacy and enhance progression-free and overall survival.

[NCT03068663] A study grouped 40 patients with NSCLC, 20 patients were treated with surgery only, while the other half also received chemotherapy. The main aim was to explore changes in the lung, upper respiratory tract and intestinal microbiota and potentially find an association between the flora and the prognosis of patients treated. [NCT06221800] Another study was conducted to collect data on the dynamics of the gut microbiome of 82 subjects with advanced/metastatic NSCLC (NSCLC) during treatment with NSCLC. The subjects were classified into 3 groups: anti-PD-1 monotherapy, anti-PD-1 combination chemotherapy and TKIs. therapy, anti-PD-1 combination chemotherapy therapy and TKIs therapy and analyzed the diversity and composition of the gut microbiome of the subjects during the treatment of NSCLC to provide reference for the efficacy of NSCLC treatment [NCT05669846]. Suitable patients will be identified at progression on PD-1 monotherapy or PD-1-containing regimens and patients will undergo a 35-day screening assessment. Following enrolment, patients will be serum matched to suitable donors and patients will receive R-FMT (induction) via colonoscopy on C1D1 and C3D1. R-FMT (maintenance) by sigmoidoscopy on C4D1 will be repeated every 9 weeks. All patients will receive an additional 200mg of pembrolizumab every 3 weeks and patients will be treated until disease progression or intolerance of toxicity or completion of 2 years of treatment (**Table 3**, **Figure 3**).

**Table 3:** Therapeutic and prognostic application potential in NSCLC patients.

NCT numbers	Title	Conditions	Intervention	Enrollment	Outcome message
NCT 06613308	Association Between Microbiome and the Efficacy and Safety of PD-1/PD-L1 Blockade in Resectable NSCLC	NSCLC (Stage IIA-IIIB)	Neoadjuvant Immunotherapy combined with chemotherapy and Neoadjuvant chemotherapy	20	respiratory and gut microbiome samples relations between respiratory and gut microbiome
NCT 03068663	Microbiota and the Lung Cancer (MICA)	NSCLC	chemotherapy and surgery (Pct-chir). surgery (Pchir).	40	samples of blood, saliva, feces, lung/tumor tissue, and bronchoalveolar lavage fluid.
NCT 05027165	Prospective Evaluation of Immunological, Molecular-genetic, Image-based and Microbial Analyzes to Characterize Tumor Response and Control in Patients With Inoperable Stage III NSCLC Treated With Chemoradiotherapy Followed by Consolidation Therapy With Durvalumab	NSCLC	15 fractions of radiotherapy	40	Stool and Blood Sample Bank for Patients
NCT 06221800	Assess Diversity of Gut Microbiome in Met NSCLC in Correlation to Tx & Adverse Effects	NSCLC	Treatment with PD1/L1 monotherapy with PD1/L1 and chemotherapy and with Tyrosine Kinase Inhibitor	82	Stool and saliva samples
NCT 05037825	The Gut Microbiome and Immune Checkpoint Inhibitor Therapy in Solid Tumors (PARADIGM)	NSCLC, MM, RCC, and TNBC; any stage	anti-PD-1, anti-PD-L1, or anti-CTLA-4 as a single agent or in combination with another checkpoint inhibitor or other treatment agent	800	Microbiome samples and Blood samples
NCT 04638751	ARGONAUT: Stool and Blood Sample Bank for Cancer Patients	advanced-stage cancer	checkpoint inhibitor therapy for the first time.	5000	Stool and Blood Sample Bank for Patients
NCT 04291755	Development and Analysis of a Stool Bank for Cancer Patients	Cancer	Any checkpoint inhibitor	100	Stool, blood, and urine specimens
NCT 04189679	Identification of a Predictive Metabolic Signature of Response to Immune Checkpoint Inhibitors in Non-Small Cell Lung Carcinoma (METABO-ICI)	NSCLC	20 treated by an ICI in first line and 40 treated by an ICI in second and third line	60	Immune signature in serum associated to the metabolic signature Meta-genomic signature of intestinal flora
NCT 04682327	Gut Microbiota and Cancer Immunotherapy Response	NSCLC	—	50	Blood samples and stool samples.
NCT 04333004	Analysis of Gut Microbiota in Patients With Brain Metastasis of Non-small Cell Lung Cancer Treated by Pembrolizumab Combined With Chemotherapy	NSCLC	Pembrolizumab Combined With Chemotherapy	40	Species and abundance of gut microbiota
NCT 04136470	BioForte Technology for in Silico Identification of Candidates for a New Microbiome-based Therapeutics and Diagnostics	NSCLC	Routine immunotherapy	100	Collection of stool, blood (PBMC) and biopsy (FFPE)
NCT 05008861	Gut Microbiota Reconstruction for NSCLC Immunotherapy	NSCLC	FMT	20	analyze the effect of FMT on intestinal flora and immunophenotype of patients.
NCT 05669846	Responder-derived FMT (R-FMT) and Pembrolizumab in Relapsed/​Refractory PD-L1 Positive NSCLC	NSCLC	FMT with Pembrolizumab	26	OS, PFS and CD8+ TIL and intra-tumoral myeloid cell density
NCT 04698161	Establishment of the Human Intestinal and Salivary Microbiota Biobank - Oncologic Diseases (BIOMIS-Onco)	NSLSC	—	50	Biological sample collection, Questionnaire and Medical examination

These clinical trials involve large cohorts and feature robust experimental design, with open results providing valuable insights into the relationship between gut microbiota, treatment efficacy, and patient outcomes. For further details and updates, *clinicaltrials.gov* is a useful resource.

## 6 Conclusion and future directions

As the importance of gut microbiota in disease development and treatment continues to be unveiled, increasing attention is being directed toward the role of gut microbiota and its metabolites in NSCLC therapy (123). NSCLC patients who achieve long-term survival exhibit distinct gut microbiota compositions. Patients with favorable prognoses typically possess a more diverse and abundant gut microbiome, and this diversity is closely linked to the activation of antitumor immune responses. For instance, high abundances of *Bacteroidetes*, *Firmicutes*, and certain lactobacilli are associated with improved immune responses and prolonged survival in NSCLC patients (18, 74). Conversely, a high abundance of *Proteobacteria* is associated with reduced efficacy of immunotherapy, and Helicobacter pylori seropositivity correlates with poorer survival in NSCLC patients receiving anti-PD-1 therapy (124).

Exogenous interventions hold promise in elucidating the complex mechanisms by which gut microbiota influence NSCLC treatment outcomes. First, drugs can induce dysbiosis by altering gut barrier function, thus affecting the efficacy of cancer treatments cancer therapy research (125, 126). Furthermore, preliminary studies on probiotics, fecal microbiota transplantation (FMT), and specific engineered strains have demonstrated potential to enhance the response to immunotherapy in NSCLC patients (127). FMT, by reintroducing beneficial gut microbiota, offers hope for treating NSCLC patients who are resistant to conventional therapies. Despite the challenges in standardizing FMT protocols, and the inherent individual variability in microbiome composition and response to interventions, the ethical considerations surrounding microbiome-based therapies require significant advancements. The intervention of gut microbiota remains in the research phase, necessitating further clinical trials to validate its efficacy (128). As such, exploring how to manipulate gut microbiota composition to achieve more efficient and less toxic treatment strategies remains a crucial direction in cancer treatment research.

The relationship between gut microbiota and treatment outcomes is multifactorial and complex. Current research primarily focuses on macro-level microbiome composition, lacking in-depth investigation into specific microbial populations and their metabolites. To date, accurately tracking dynamic changes in the microbiome remains challenging, and a deeper understanding of the mechanisms by which specific microbes influence therapy is needed. This review primarily focuses on metagenomic sequencing, metabolomic profiling, and 16S rRNA sequencing to explore the specific roles of gut microbiota in NSCLC treatment. Future research should integrate finer techniques, such as single-cell sequencing and spatial transcriptomics, to establish connections from the disease and immune microenvironment perspective (129, 130). Ultimately, gut microbiota intervention is expected to become a routine component of NSCLC treatment, providing new therapeutic avenues for cancer immunotherapy, chemotherapy, and targeted therapy. With advancements in technology and the progression of clinical trials, we are optimistic that gut microbiota will become a crucial factor in NSCLC treatment, driving cancer therapy towards more personalized and precision-based approaches.
